# Fabrication and evaluation of a BMP-2/dexamethasone co-loaded gelatin sponge scaffold for rapid bone regeneration

**DOI:** 10.1093/rb/rbac008

**Published:** 2022-02-16

**Authors:** Qi Gan, Hao Pan, Wenjing Zhang, Yuan Yuan, Jiangchao Qian, Changsheng Liu

**Affiliations:** 1 Key Laboratory for Ultrafine Materials of Ministry of Education, East China University of Science and Technology, Shanghai 200237, PR China; 2 The State Key Laboratory of Bioreactor Engineering, East China University of Science and Technology, Shanghai 200237, PR China; 3 Engineering Research Center for Biomedical Materials of the Ministry of Education, East China University of Science and Technology, Shanghai 200237, PR China; 4 Frontiers Science Center for Materiobiology and Dynamic Chemistry, East China University of Science and Technology, Shanghai 200237, PR China

**Keywords:** BMP-2, dexamethasone, Runx2, pre-clinical, bone regeneration

## Abstract

Improving the osteogenic activity of BMP-2 *in vivo* has significant clinical application value. In this research, we use a clinical gelatin sponge scaffold loaded with BMP-2 and dexamethasone (Dex) to evaluate the osteogenic activity of dual drugs *via* ectopic osteogenesis *in vivo*. We also investigate the mechanism of osteogenesis induced by BMP-2 and Dex with C2C12, a multipotent muscle-derived progenitor cell. The results show that the gelatin scaffold with Dex and BMP-2 can significantly accelerate osteogenesis *in vivo*. It is indicated that compared with the BMP-2 or Dex alone, 100 nM of Dex can dramatically enhance the BMP-2-induced alkaline phosphatase activity (ALP), ALP mRNA expression and mineralization. Further studies show that 100 nM of Dex can maintain the secondary structure of BMP-2 and facilitate recognition of BMP-2 with its receptors on the surface of C2C12 cells. We also find that in C2C12, Dex has no obvious effect on the BMP-2-induced Smad1/5/8 protein expression and the STAT3-dependent pathway, but Runx2-dependent pathway is involved in the Dex-stimulated osteoblast differentiation of BMP-2 both *in vitro* and *in vivo*. Based on these results, a potential mechanism model about the synergistic osteoinductive effect of Dex and BMP-2 in C2C12 cells *via* Runx2 activation is proposed. This may provide a theoretical basis for the pre-clinical application of Dex and BMP-2 for bone regeneration.

## Graphical Abstract

**Figure:**
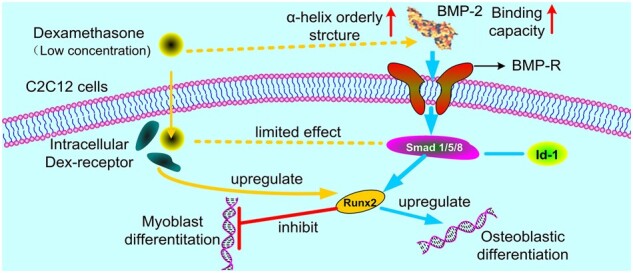

## Introduction

Large bone defects and non-unions resulting from resection, trauma or pathology are serious public health problems and common clinical problems, especially for the elderly [1]. However, currently used orthopedic implants show unsatisfactory therapeutic effects motivating the fabrication of more effective bone substitutes [2–4]. To address this, the addition of growth factor is an attractive strategy. As a member of the transforming growth factor (TGF-β) superfamily, bone morphogenetic protein-2 (BMP-2) has been considered to be the most effective growth factor that can promote osteogenesis and bone tissue remodeling [5–8]. In 2002, BMP-2 was approved by the US FDA and European Medicines Agency for clinical use, including spinal fusion, fracture healing and implant fixation. Unfortunately, the clinically required high BMP-2 level and thereby possible side effects, such as induction of bone resorption, abnormal osteogenesis and nerve cell responses in non-repair areas greatly affect the wide application of BMP-2 in clinic [9–12]. Urgently, it calls for a strategy to promote the osteoblastic bioactivity of BMP-2.

As a matter of fact, various approaches have been attempted from different perspectives to enhance/promote the biological activity of BMP-2 *in vivo* and *in vitro*. Nowadays, formulating the growth factor into micro- and nano-carrier delivery systems may represent the most commonly used method to maintain protein stability and prolong the therapeutic effect [13, 14]. However, in clinical applications, the BMP-2 carriers that can be used are just several mature products, such as gelatin sponge and calcium phosphate cement (CPC) [15]. Recently, combined use with a positive modulator of BMP-2, such as the cytokines, synthetic glucocorticoids, hormone and vitamin, may be an appealing strategy for clinical application [16–20]. Among these, non-growth factor dexamethasone (Dex), which is considered to be one of the earliest and most widely used inducers of bone-related cells differentiation, appears to be most attractive [21–24]. Previous investigations have acclaimed that in BMSCs, Dex with appropriate dosage could effectively enhance the osteogenic differentiation induced by BMP-2 [23, 25]. These studies have strongly proven the feasibility to enhance the BMP-2-induced osteogenic differentiation and bone mineralization by Dex and thus lower the dosage of BMP-2 through their synergistic efficacy. However, the role of Dex in BMP-2-induced bone formation is still not clear. Especially, the mechanism related to two widely used methods for evaluation of the bioactivity of BMP-2, which are the ectopic bone formation and incubation with C2C12 cells still need enormous research.

In this study, we implanted a clinical gelatin sponge scaffold loaded with BMP-2 and Dex into the muscle bags of mice, and found the ectopic bone formation was significant *in vivo*. Furthermore, C2C12 cell line was chosen to explore the effect of Dex on the capacity of BMP-2-inducing osteoblastic differentiation and bone mineralization. Alkaline phosphatase activity (ALP), a typical osteoblast-specific marker, was used to quantify the activity of osteoblastic differentiation. Both the dose- and time-dependent effects of Dex on the biological activity of BMP-2 were studied *in vivo* and *in vitro*. We also research the effect of Dex on the secondary structure of BMP-2 and the binding capacity of BMP-2 with its receptors on C2C12 cell membrane. Meanwhile, we also investigated the effects of Dex on Smad-signaling, signal transducer and activator of transcription 3 (STAT3) dependent mechanism, *in vitro* mineralization and effects of Dex and BMP-2 on the expression of osteogenic markers and related transcription factors, in order to propose the potential mechanism about the synergistic osteoinductive effect of Dex and BMP-2 in C2C12 cells.

## Materials and methods

### Materials

Dex, *p*-nitrophenyl phosphate (PNPP-Na), FITC-Phalloidin and Alexa Fluor 647-linked goat-anti-mouse IgG were all purchased from Sigma-Aldrich (CA, USA). Gelatin sponge scaffolds were purchased from Jingling Med Co. Ltd (Nanjing, China). rhBMP-2 protein was purchased from Shanghai Rebone Co. Ltd (*Escherichiacoli*-derived, China). The antibody for anti-BMP-2 was obtained from R&D systems Co. Ltd (USA). Bicinchoninic acid (BCA) Assay Kits and DAPI were obtained from Biyuntian Biological Co. Ltd (Suzhou, China). Reagents related to cell culture were all obtained from Gibco Company (New York, USA).

### Ectopic bone formation *in vivo*

This study followed the NIH laboratory animals care and was approved by the Laboratory Animal Center of National Tissue Engineering Research Center. Dex (0, 4, 20 and 100 μg) and 15 μg of BMP-2 were added onto gelatin sponge scaffolds (5 × 5 × 3 mm), respectively, then freeze-dried at low temperature, and stored at −20°C. A total of 24 male mice (1 month old, Shanghai Slack Company, China) were anesthetized with diethyl ether inhalation. Animal mice were divided into four sample groups, and gelatin sheets were implanted in the muscle pocket of the right thigh. Two weeks after the operation, the mice were sacrificed by cervical vertebrae and the implanted samples were obtained. A micro-CT scanned image (eXplore Locus SP) was used to evaluated the bone formation. All scans use 80 kV voltage and 80 mA current. The projected image was taken over 360° in 0.4° increments. GE Microview ABA 2.1.2 software was used to carry out bone radiation morphometric analysis. In the Region of Interest of each set part, we further quantitatively detect the volume of new bone and tissue mineral density.

The *in vivo* Runx2 protein was detected as the below-mentioned Western blotting analysis in this experiment. Two weeks after implantation, the implants were harvested and then lysed for 30 min with RIPA Lysis Buffer containing 1.0 mM phenylmethanesulfonyl fluoride. The total tissue protein lysis buffer was centrifuged at 1.2 × 10^4^ rpm for 5 min and the supernatant was collected. Then, the rest steps were similar to the determination of p-Smads/Runx2 proteins *in vitro* in ‘Western blotting analysis’ section.

### Cell culture

The C2C12 cell, a mouse myoblast cell line with osteoblastic potential, was obtained from the American Type Culture Collection. C2C12 cells were cultured in a 37.5 cm^2^ flask with DMEM containing 10% fetal bovine serum (FBS), antibiotics (100 mg/ml streptomycin and 100 U/ml penicillin-G) (we called growth medium) in a 5% CO_2_/95% air incubator, digest and separate with 0.25% trypsin/0.03% ethylenediaminetetraacetic acid. The cells were counted and used in subsequent experiments.

### Measurement of ALP activity

As mentioned earlier [26], ALP activity was measured with PNPP-Na. ALP activity was set as the absorbance value at 405 nm per milligram of protein per minute. Use BCA kit (Beyotime, Jiangsu, China) to detect the total protein concentration of cells, with bovine serum albumin (BSA) as the standard. In order to determine the ALP activity by histochemical method, the cells were fixed, then washed with PBS for several times, and incubated with a mixture with 1 mg/ml Fast Blue BB salt (Shanghai Yuanju, China), 0.5 mg/ml naphthol AS-BI phosphate (Sigma, CA, USA), 0.05 mol/l MgCl_2_, 0.01 mol/l levamisole and 2% (V/V) dimethyl formamide (DMF) for 30 min at 37°C. The fixed cells were imaged with a Leica DMi8 optical microscope (Leica, Germany).

### Mineralization assay

For determining the formation of bone nodules, the mineralization of extracellular matrix was indicated by Alizarin Red S staining as previously described [27]. Cells were cultured in maintenance medium (containing 2% FBS) for 2 or 3 weeks, and the cell medium was changed every 2 days. Subsequently, within the set time, the cells were washed twice with PBS solution at room temperature (RT) and fixed with 4% paraformaldehyde (w/v) solution for 15 min. We then stained the fixed cells with 1% Alizarin Red (Shanghai, China) (pH = 4.2) for 15 min. Then, we washed the cells with PBS for twice, and digital images of the stained cells were observed and taken with an optical microscope.

### Binding capacity of BMP-2 to the C2C12 cell membranes

We performed the immunofluorescence assay to analyze the binding efficiency of BMP-2 to the cell membranes. C2C12 cells were adhered for 24 h and then treated with elevated Dex in the presence of 0.4 μg/ml BMP-2 for 6 h. After that, we fixed the cells with 4% paraformaldehyde and blocked the n-specific binding sites with 5% FBS/PBS solution at RT for 1 h. After that, we incubated the cells with anti-BMP-2 antibody (R&D, USA) for another 1 h at RT followed by Alexa Fluor 647-linked goat-anti-mouse IgG (Sigma-Aldrich, USA) for staining BMP-2. The cytoskeleton was stained with FITC-Phalloidin (Sigma, USA) and the nuclei were stained with DAPI solution at RT. We used a confocal microscope (A1R, Nikon, Japan) to measure BMP-2, cytoskeleton and nucleus at 562, 488 and 405 nm wavelengths, respectively.

### Far-ultraviolet circular dichroism detection

The secondary structure of BMP-2 affected by Dex was measured by far-ultraviolet circular dichroism (far-UV CD) spectroscopy (Model J-715, Japan). The far-UV CD spectrum was obtained at a wavelength of 190–260 nm. We used a rectangular quartz cell with 1 mm path length for testing, and used a time constant of 250 ms at a scan rate of 1 0^−7^ m/min. Each spectrum data was tested for three times and the average value was taken. CDNN Deconvolution Software (version 2.1) was used to calculate the secondary structure of BMP-2.

### RT-PCR analysis

In the presence of the specified concentration of the inducer, the cells were grown to 80% confluency in a 6-well plate with maintenance medium. According to the instructions of real-time reverse transcription polymerase chain reaction, total RNA was extracted using a total RNA Extraction Kit (Axygen, USA). We synthesized the first strand cDNA with PrimeScript RT Reagent kit (Takara, Japan). After that, we diluted the cDNA 10-fold with sterile distilled water, and then subjected a 4 μl diluted cDNA to real-time polymerase chain reaction (RT-PCR) using SYBR PCR Kit (Takara, Japan). RT-PCR was performed in a 10 μl mixed solution, which was composed of 1 ×  polymerase chain reaction (PCR) buffer, 1 × SYBR Premix Ex Taq (5 μ), 4 μl aliquot of the diluted cDNA, 0.2 μl ROX Reference Dye, 0.4 μl of forward primer and 0.4 μl of reverse primer. The experimental conditions of real-time fluorescence quantitative PCR were like these: raised to 95°C and maintained for 30 s, then, run for 40 cycles, 95°C for 5 s and 60°C for 34 s. The result of RT-PCR was detected by ABI 7900HT instrument. The primer sequences used in this study were shown in Table 1.

**Table 1.** Sense and antisense primers utilized for RT-PCR amplification

Target	Forward primer sequence	Reverse primer sequence
ALP	5′-CCA ACT CTT TTG TGC CAG AGA-3′	5′-GGC TAC ATT GGT GTT GAG CTT TT-3′
Runx2	5′-CGG CCC TCC CTG AAC TCT-3′	5′-TGC CTG CCT GGG ATC TGT-3′
Osteocalcin	5′-CTG ACA AAG CCT TCA TGT CCA A-3′	5′-GCG GGC GAG TCT GTT CAC TA-3′
Id-1	5′-GCG CAC CGT GAA CCT AAA C-3′	5′-GTG GAC TGG CAA TGG AGA AAC-3′
Collagen I	5′-GGT ATG CTT GAT CTG TAT CTG C-3′	5′-AGT CCA GTT CTT CAT TGC ATT-3′
GAPDH	5′-GTC GTG GAG TCT ACT GGT GTC-3′	5′-GAG CCC TTC CAC AAT GCC AAA-3′

### Western blotting analysis

C2C12 Cells were incubated with rhBMP-2 (Rebone, China), Dex and a combination of two inducers for 4 h to determine the phospho-Smad1/5/8 protein, while treated for 72 h to determine the Runx2 protein. C2C12 cells were lysed with 1× lysis buffer, and then determine the total protein concentration using BCA detection kit with BSA as the standard. The obtained protein sample (10 μg) was separated and transferred to a membrane. Then, it was blocked with 5% skimmed milk, and then incubated with a diluted solution (1:500) including anti-phospho Smad1/5/8 and anti-actin (Santa Cruz, CA) at 4°C for 12 h. After that, the membrane was washing with water and incubated with the secondary antibody for 1 h. The bands were displayed by chemiluminescence according to the manufacturer’s instructions.

### Statistical analysis

All data provided here are in the form of mean ± standard deviation, and similar results were obtained in each of our experiments. Statistical analysis is using one-way analysis of variance. A value of *P* < 0.05 is considered statistically significant.

## Results and discussion

### Evaluation of ectopic osteogenesis of BMP-2+dex@gelatin scaffold *in vivo*

**Figure 1.**
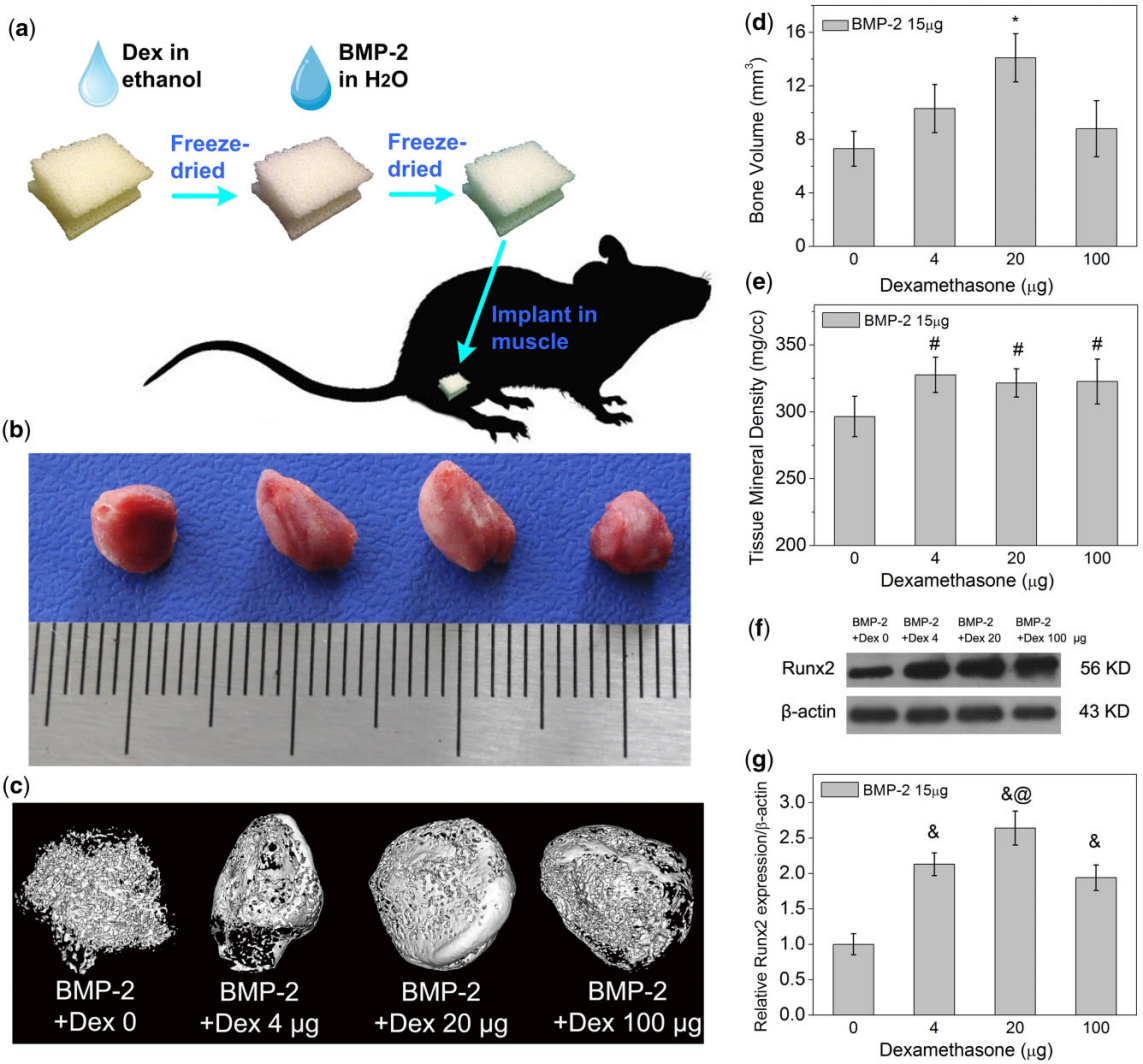
Effect of Dex on BMP-2-induced ectopic osteogenesis *in vivo*. (**a**) Gelatin sponge scaffold with 15 μg of BMP-2 and 0, 4, 20, 100 μg Dex was implanted into thigh muscle pouches of mice to stimulate ectopic osteogenesis. The digital pictures (**b**) and micro-CT images (**c**) of new ectopic bone after 2 weeks of surgery were displayed (*n* = 6). Use GE software-Microview ABA 2.1.2 to quantitatively analyze the new bone volume (**d**) and tissue mineral density (**e**) from the micro-CT image. The data presented are the average of three independent experiments. (**f**) Western blot analysis of Runx2 and β-actin protein abundances in ectopic bone formation. (**g**) Integrated *in vivo* Runx2 optical data analysis by ImageJ 1.42. * indicates that group with BMP-2 and 20 μg Dex contained higher bone volume than groups without Dex. # indicates that the tissue mineral density of groups with Dex and BMP-2 all higher than groups without Dex. & indicates that the Runx2 protein levels *in vivo* with Dex and BMP-2 higher than BMP-2 group. @ indicates that the Runx2 protein expression of the group with BMP-2 and 20 μg Dex is significantly higher than the other groups. *P, *#P*, *&P*, *@P *<* *0.05

We examined the effect of gelatin sponge scaffold with Dex and BMP-2 (BMP-2+Dex@gelatin) on the ectopic osteogenesis using the mouse thigh muscle model *in vivo* (Fig. 1a). In preliminary experiments, we found that 15 μg BMP-2 per mice could effectively induce the ectopic bone formation and 1–150 μg of Dex could promote the process *in vivo*. So, in this study, we fixed the dosage of BMP-2 at 15 μg per mice and Dex within 4–100 μg. Within 2 weeks, in the presence of 20 μg of Dex, the new bone formation was significantly increased than groups without Dex (Fig. 1b). The blank group without BMP-2 had no ectopic bone formation (data not shown). The detection of μCT could distinguish the mineralized tissue from the soft tissue remaining in the implant. It revealed that the compaction of mineralized tissues of Dex-loaded groups was slightly better than control group (Fig. 1c). We also calculated the volume of new bone (Fig. 1d) and tissue mineralization density (Fig. 1e) to more accurately assess the condition of ectopic bone formation. The Runx2 protein expression *in vivo* was measured by western blotting analysis (Fig. 1f and g). The expressions of Runx2 proteins *in vivo* with Dex and BMP-2 are both higher than BMP-2 group, meanwhile, the group with BMP-2 and 20 μg Dex shows the highest Runx2 protein expression. From our animal results, it was demonstrated that a gelatin sponge scaffold with appropriate concentration of BMP-2 and Dex could enhance bone formation *in vivo*. With relative low amount of Dex (20 μg/implantation), BMP-2 induced higher value of bone volumes and tissue mineral density within 2 weeks in mice.

### Effect of Dex and BMP-2 on osteoblastic differentiation *in vitro*

**Figure 2.**
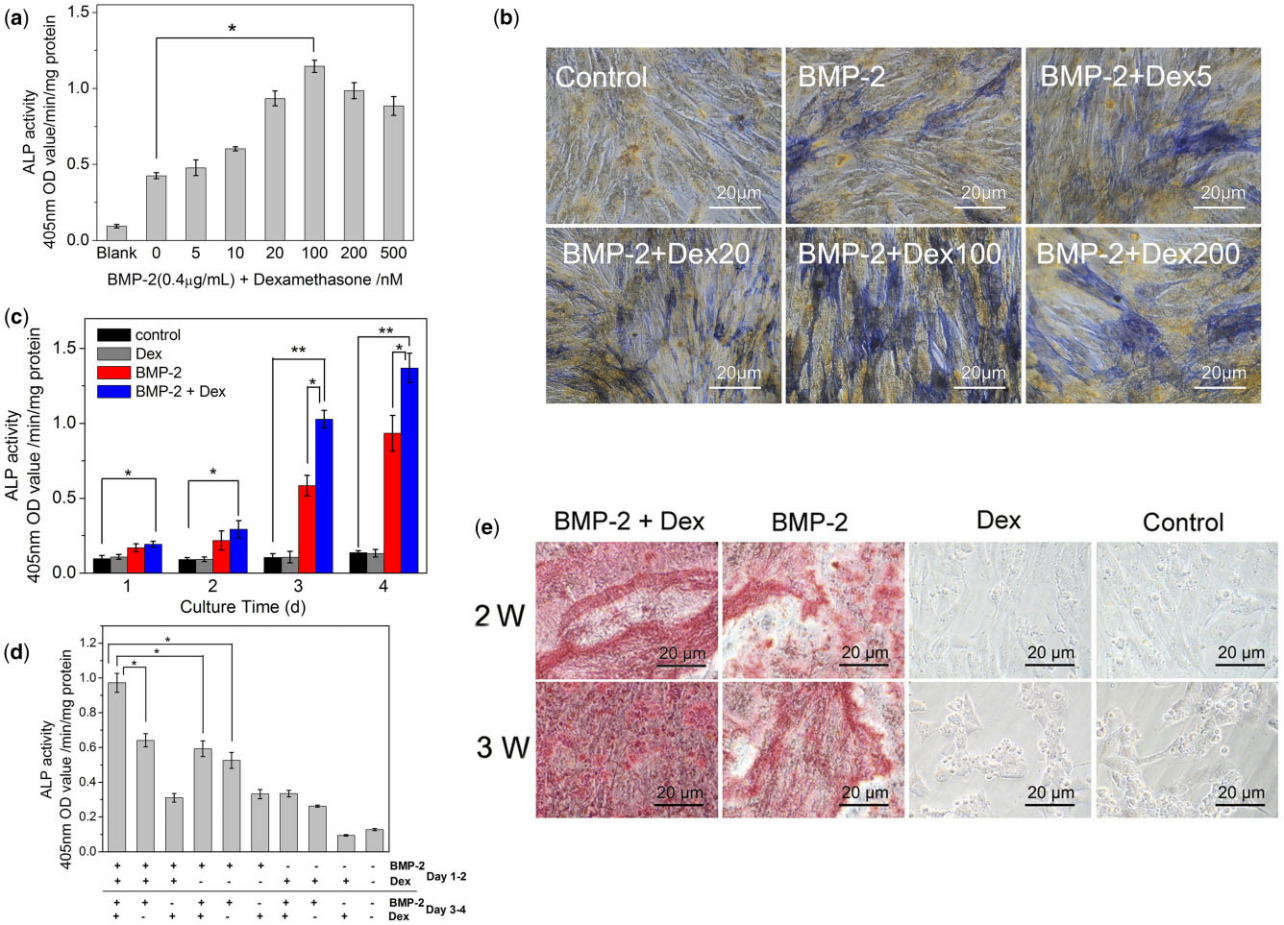
Dex enhanced the biological activity of BMP-2. (**a**) ALP was measured after cells were cultured with 0.4 μg/ml of BMP-2 and 0, 5, 10, 20, 100, 200 or 500 nM of Dex for 3 days. The data were obtained from the color reaction of *p*-nitrophenylphosphate. (**b**) The staining of ALP activity was using fast blue BB and naphthol as-BI phosphate salt. (**c**) ALP activity of C2C12 cells for different incubation times. Cells incubated with BMP-2 (0.4 μg/ml), Dex (100 nM) or a combination of both for different incubation times. (**d**) The ALP activity was induced by adding inducers at different time periods. For the first 2 days of C2C12 cell culture, used BMP-2 (0.4 μg/ml), Dex (100 nM) or a mixture of two inducers as the medium, and removed the inducer after 48 h of culture. Then, we cultured the cells with the designated inducers from Day 3 to 4. ALP activity was measured at Day 4 (*n* = 4). (**e**) Effects of BMP-2 and Dex on C2C12 cells mineralization. The culture medium of C2C12 cells were BMP-2 (0.4 μg/ml), Dex (100 nM) or a mixture of two inducers. The staining of cell mineralization was detected using 1% Alizarin Red S and checked at 400× magnification. *P <* *0.05. **P <* *0.001

Before determining the effect of Dex on the BMP-2-induced ALP levels, we first treated C2C12 cells with BMP-2 or Dex alone at different concentrations for 3 days. When the concentration of BMP-2 was equal to or bigger than 0.4 μg/ml, it could be observed that the ALP activity had increased significantly. But in the case of cells treated with Dex alone, the expression of ALP did not show statistically significant difference compared to that without any inducer (Supplementary Figs S1 and S2). The dose–dependence of Dex on the BMP-2-induced osteoblastic differentiation was detected subsequently. It can be seen that BMP-2 and 5–200 nM of Dex all expressed higher ALP activity compared to samples treated with BMP-2 alone (Fig. 2a). Specifically, the ALP levels monotonically increased with the increasing of Dex concentration up to 100 nM, and then slightly decreased or remained constant afterwards. Figure 2b shows that after the culture, the staining of cells treated with (0.4 μg/ml BMP-2 + 100 nM Dex) was the most intensely.

The culture time-dependent effects of the two inducers on ALP expression were detected (Fig. 2c). The cells simultaneously treated with both Dex and BMP-2 showed the highest ALP activity at all indicated times. To clarify whether Dex and BMP-2 affect the differentiation of C2C12 cells at different stages, the results in Fig. 2d show a comparison of the relative ALP expression in the ‘early treatment’ (1–2 days) and ‘late treatment’ (3–4 days) cells. As can be seen, treatment with the combination of Dex and BMP-2 in the early and late stages induced the highest ALP expression. The mineralization staining checked by Alizarin Red is shown in Fig. 2e. Cells treated with a combination of Dex and BMP-2 or BMP-2 alone showed more stained areas, indicating the formation of mineralized products, than that treated with Dex or control (no inducer), at both 2 and 3 weeks.

According to the previous literature [28], BMP-2 was considered to have greater osteogenic differentiation ability in pre-osteoblasts or pluripotent stem cells, but the osteogenic differentiation effect was poor in mature osteoblast cell lines. For osteoblasts, Dex has favorable and unfavorable effects on the cell mineralization and osteoblast differentiation, which depending on the concentration of Dex, cell type and cell density [29–31]. Together with our results that Dex did not affect the C2C12 cells’ ALP expression, it could be hypothesized that the promoting effect of Dex and BMP-2 on osteoinductive activity might takes place *via* mediating BMP-2-induced osteogenesis. However, in C2C12 cells, our results showed that continuous treatment with any inducer alone showed better results than switching from one inducer to another (Fig. 2d). And the role of BMP-2 was very important in both ‘early-treated’ and ‘later-treated’ periods. These results indicated that in C2C12, Dex and BMP-2 might be acting in the same differentiation stage or the same pathway. Inconsistent with the previous results, both Dex and BMP-2 can be used as osteoblast stimulators in human BMSCs, but show different effects at different stages of osteoblast differentiation [28]. Furthermore, Dex seems to be more effective in the BMP-2-mediated osteoblast differentiation in equine BMSCs, whereas less effective in human BMSCs [32]. It was indicated that the effects of Dex on the BMP-2-induced osteoblast differentiation may be cell type and concentration dependent.

### Evaluation of the binding capacity and secondary structure of BMP-2 incubating with Dex

**Figure 3.**
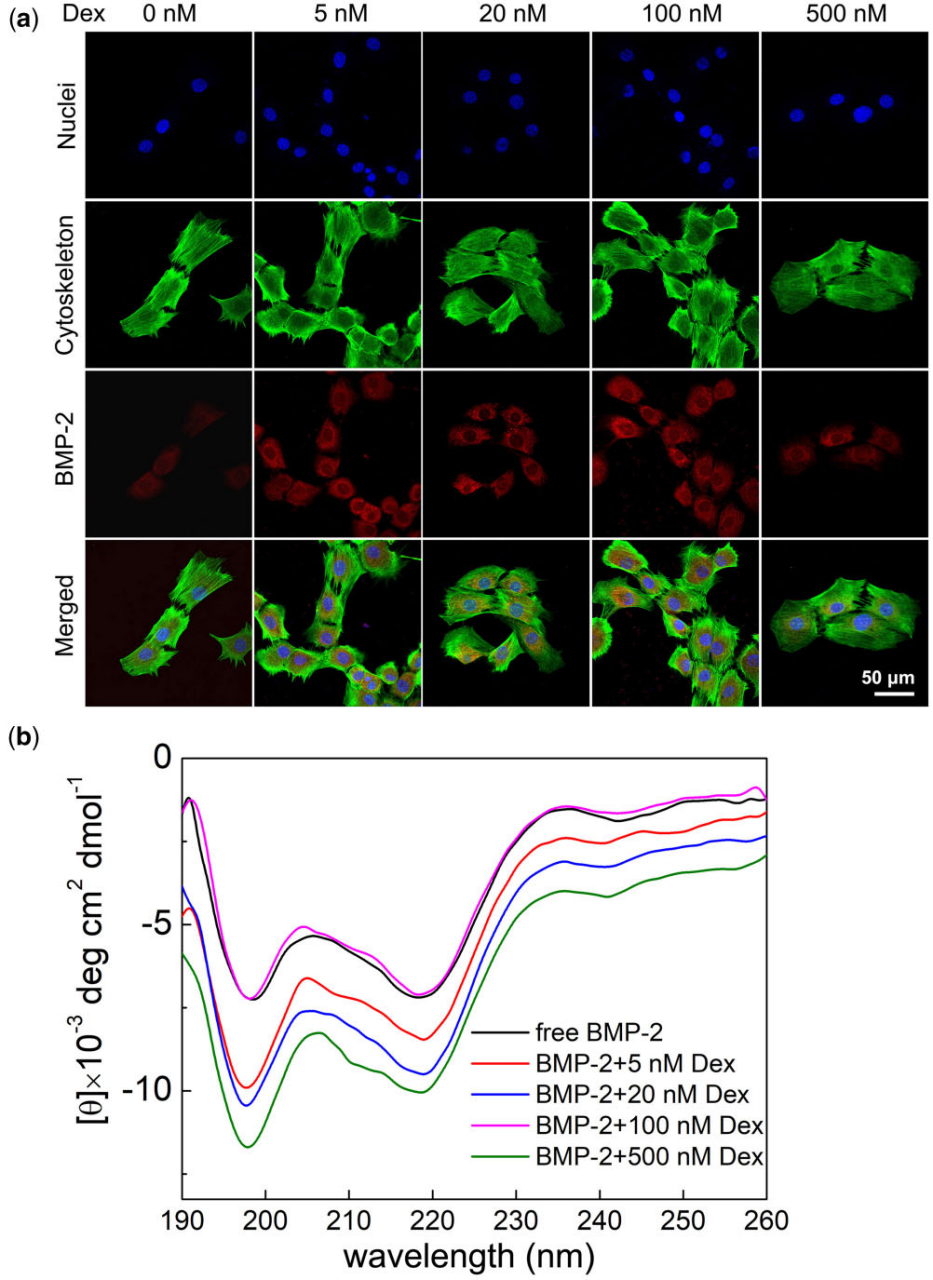
(**a**) Effect of Dex on the binding of BMP-2 to C2C12 cell membranes. The cells were incubated with 0.4 μg/ml BMP-2 and different concentrations of Dex for 6 h. FITC-labeled phalloidin (green) was used to stain the cell cytoskeleton and DAPI (blue) was used to stain the nuclei. BMP-2 on the cell surface was detected with alexa fluor 647-labeled anti-mouse IgG (red) and anti-BMP-2 antibodies. (**b**) Far-UV CD spectrum of BMP-2 at different concentrations of Dex. Secondary structure of BMP-2 was detected with related software

Typically, the important step in BMP-2-mediated osteogenic differentiation is to make BMP-2 bind to its receptors. In our research, the binding of BMP-2 to cell membrane surface receptors was evaluated by immunocytochemistry (Fig. 3a). Despite Alexa Fluor 647-labeled BMP-2 was detected on the cell membranes of all samples after 6 h of incubation, the fluorescence intensity increased when the concentration of Dex was 5–500 nM, and the highest fluorescence intensity was observed for 100 nM Dex. It was indicated that less Dex enhanced the binding of BMP-2 to BMP-2 receptor (BMP-R) on the cell surface, while high concentration of Dex would reduce the binding efficiency of BMP-2 to its receptors.

Figure 3b shows the far-UV CD spectra of BMP-2 in the presence of Dex with different concentrations. The secondary structural elements of free BMP-2, 0.4 μg/ml BMP-2 with 5, 20, 100 and 500 nM Dex in 0.5 mM PBS were exhibited in Table 2. For free BMP-2, the contents of alpha-helix (α-helix), beta-sheet (β-sheet), beta-turn (β-turn) and random coil were 16.6%, 15.67%, 15.46% and 52.27%, respectively. With progressive addition of Dex, the percentages of α-helix, β-sheet, β-turn, and random coil were all slightly changed. The percentage of α-helix was increased by 0.37% in BMP-2 + 100 nM Dex, while for BMP-2 + 5-, 20- and 500-nM Dex, the percentages of α-helix were decreased by 1.73%, 0.49% and 1.65%. Compared to free BMP-2, the addition of 100 nM Dex showed the smallest changes of the folding structure.

**Table 2.** Secondary structure of free BMP-2, 0.4 μg/ml BMP-2 with 5, 20, 100 and 500 nM Dex in 0.5 mM PBS as determined by CD spectra^a^

Secondary structure compositions	Free BMP-2 (%)	BMP-2 + 5 nM Dex (%)	BMP-2 + 20 nM Dex (%)	BMP-2 + 100 nM Dex (%)	BMP-2 + 500 nM Dex (%)
α-Helix	16.60	14.87	16.11	16.97	14.95
β-Sheets	15.67	13.92	11.19	15.34	11.05
β-Turns	15.46	15.51	14.33	15.13	15.17
Rndm. coil	52.27	55.70	58.26	52.35	58.72
Changes of the folding structure		12.1	5.6	1.11	13.0

^a^ Software CDNN V2.1 was used to analyze the190–260 nm spectra region to evaluate the secondary structure of protein.

As an important member of TGF-β protein, BMP-2 initiates its function through binding to BMPR-IA/IB receptor with high affinity and subsequently BMP-II receptor with lower affinity on the cellular surface to form a signaling complex, and then activates the downstream signaling pathway (Smad, MAPK and STAT3 pathways). Therefore, from this viewpoint, when analyzing the role of Dex in regulating the osteogenic differentiation induced by BMP-2, the effect of Dex on the binding of BMP-2 to its receptor and the subsequent signal pathways should be studied. Herein, the presence of Dex in a concentration of 0–500 nM could facilitate recognition of BMP-2 with BMP-R on C2C12 cell surface. The results indicated that in the presence of Dex, the efficient combination of BMP-2 and BMPR-R played a critical role in determining the osteogenic activity of BMP-2. Moreover, it also can be hypothesized that high concentration of Dex (500 nM) might down-regulated the combination efficiency of BMP-2 and BMP-R. Similar to our results, Matsumoto [33] reported 1 μM Dex inhibited BMP-2-mediated expression of Runx2 and reduced the expression of osteocalcin (OC). Furthermore, the CD spectra showed that the concentration of Dex had a significant effect on the secondary structure of BMP-2. Thus, the osteogenic differentiation of C2C12 cells was closely related to the balance between the concentration of Dex and BMP-2.

### Effects of Dex on the signal pathway and osteoblast markers of BMP-2

**Figure 4.**
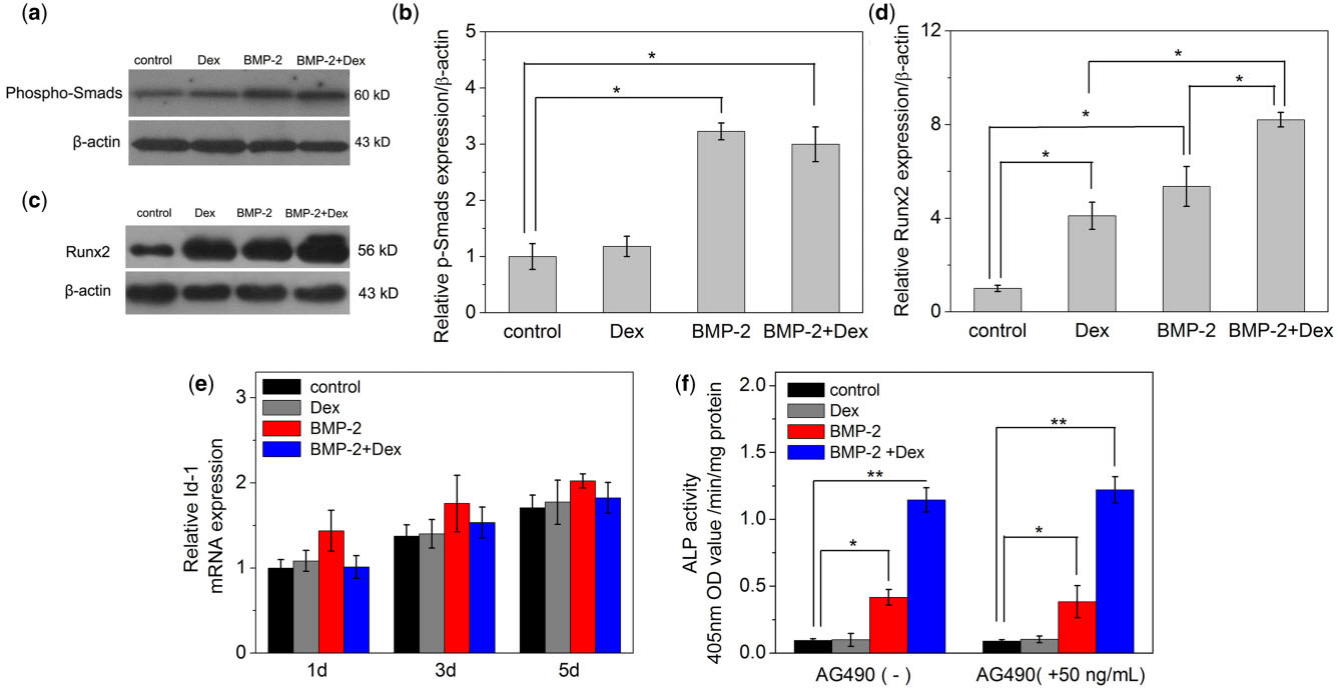
Effects of Dex on osteoblastic differentiation induced by BMP-2. (**a**) Effects of Dex on Smad1/5/8 phosphorylation signaling pathway induced by BMP-2. Western blotting for phosphorylated Smad1/5/8 was detected andβ-actin was set as the blank group. (**b**) Integrated p-Smads optical data analysis by ImageJ 1.42. (**c**) Western blot analysis of Runx2 and β-actin in C2C12 cells *in vitro*. (**d**) Integrated Runx2 optical data analysis by ImageJ 1.42. (**e**) Detection of the expression of id-1 affected by Dex and BMP-2. The mRNA expression of id-1 was measured with RT-PCR (*n* = 4). (**f**) ALP activity of C2C12 cells cultured with Dex and BMP-2 and the JAK/STAT inhibitor AG490. The concentration of BMP-2 in all experiments is 0.4 μg/ml and Dex is 100 nM. **P <* *0.001, *P <* *0.05

Figure 4a and b shows that no marked difference in phospho-Smad1/5/8 levels was found in C2C12 cells incubated with (BMP-2+Dex) or BMP-2. Moreover, we tested a targeting factor that activates Smad signaling, Id-1, and found that treated with Dex had no effect on the expression of Id-1 induced by BMP-2 (Fig. 4e). Next, the STAT3 was investigated in our research and whether it played a key role in this synergy process. We have used an inhibitor of STAT3 phosphorylation, AG490, to confirm our conjecture [22]. Figure 4f shows that there was no obvious enhancement or inhibition on ALP activities of C2C12 cells treated with inducers after adding AG490. From the previous reports, cytokines that affect the differentiation of osteoblasts included the IL6 family [34]. Janus kinases (JAKs) were activated by the binding of IL-6 to its receptor, and then STAT3 was phosphorylated by activated JAKs, thereby regulating the transcription of multiple genes and differentiation of osteoblasts. [35, 36]. With adding AG490 in culture medium, we found that STAT3 had no effect on the ALP activity of the experimental groups (Fig. 4f). Somewhat contradictory to previous report, in which they found that in C3H10T1/2 [22], a mouse embryonic fibroblast cell line, Dex and BMP-2 could significantly enhance ALP activity and accelerate early osteoblast differentiation through the STAT3 signaling pathway. We believe this difference might be related to the cell type.

**Figure 5.**
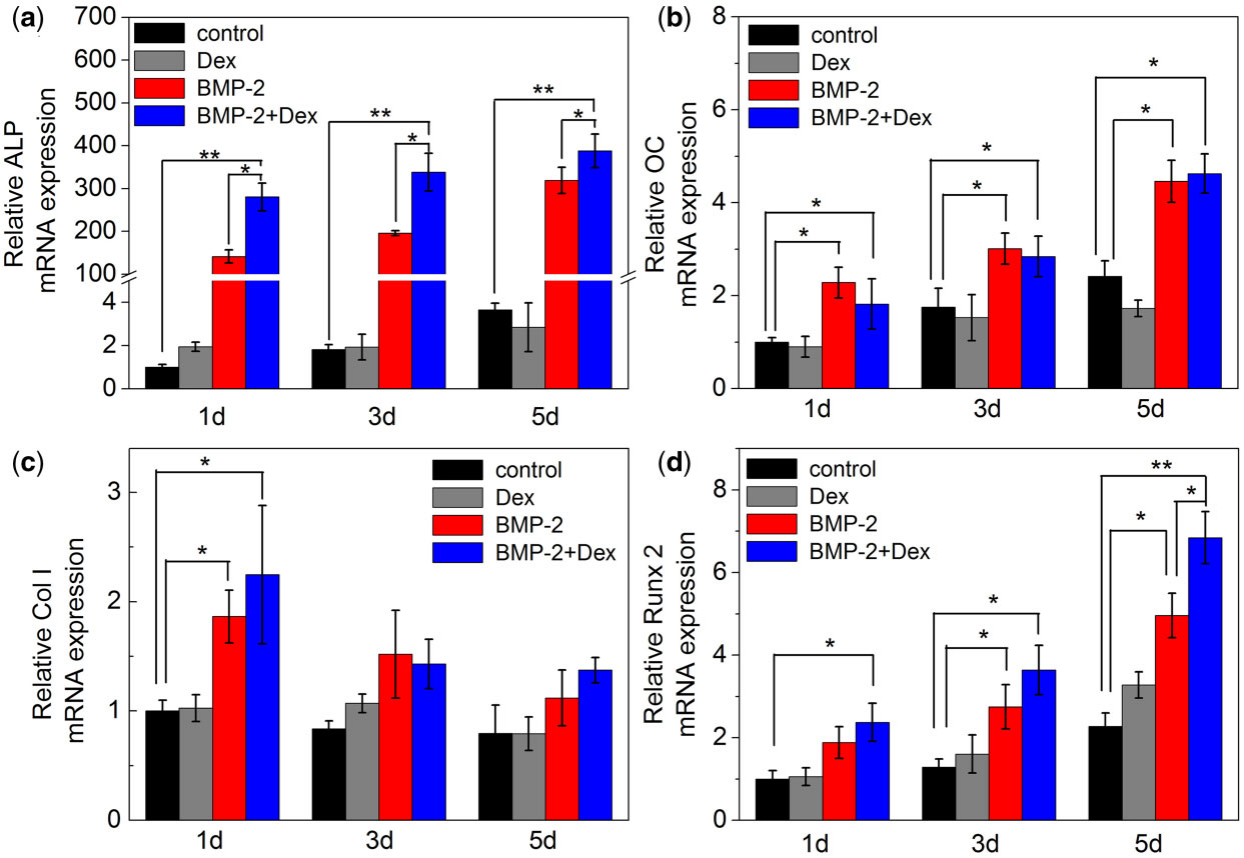
The expression of osteoblast markers and osteogenic transcription factors affected by BMP-2 and Dex, (**a**) ALP, (**b**) OC, (**c**) collagen I and (**d**) Runx2, in C2C12 cells. Cells were incubated with BMP-2 (0.4 μg/ml), Dex (100 nM) or a mixture of two inducers for 1, 3 and 5 days and characterized with RT-PCR (*n* = 4). Control indicates the cells cultured in a medium without BMP-2 and Dex. *P <* *0.05, ** *P *<* *0.001

In order to further explore whether the combined enhancement of ALP activity by Dex and BMP-2 is related to bone formation-related transcription factors, we measured mRNA levels of ALP, osteocalcin (OC), collagen I (Col I) or Runx2 after 1, 3 and 5 days treatment (Fig. 5). Treatment with (Dex+BMP-2) strongly enhanced the expression of Runx2 mRNA at 1 and 3 days, especially at 5 days (Fig. 5d). Treatment with Dex or BMP-2 alone all enhanced the expression of Runx2 at 3 and 5 days, compared to that in control group. The Runx2 protein expressions of C2C12 treated for 3 days *in vitro* exhibited similar results detected by western blots (Fig. 4c and d). From the semi-quantitative analysis, it was found that the level of Runx2 expression in cells incubated with BMP-2 or Dex alone was significantly lower than the experimental group treated with BMP-2+Dex. Apparently, Dex and BMP-2 could synergistically enhance the expression of Runx2 mRNA and protein in the indicated times.

### The potential mechanism about the regulation of osteoblast differentiation with BMP-2 and Dex

It was demonstrated that BMP-2+Dex@gelatin scaffold could enhance bone formation *in vivo* from our animal results. Furthermore, we have selected C2C12 cells as our model cell line and then investigate the effect of Dex on osteoblast differentiation induced by BMP-2 and the related mechanism *in vitro*. The presented data confirmed that Dex, as expected, could dramatically increase the ALP activity of C2C12 cells treated with BMP-2 in typical time- and concentration-dependent manners. Moreover, Dex and BMP-2 could increase the expression of Runx2 mRNA and protein both *in vitro* and *in vivo*.

In general, Activating the Smad-Runx2 signaling pathway is the way for BMP-2 to display its osteogenic activity. After BMP-2 binds to its receptor, it stimulates the phosphorylation of Smad1/5/8 and binds to Smad4. The complex interacts with Runx2 in the nucleus to activate the transcription of the genes which contain OSE2, thereby activating osteogenic differentiation [37, 38]. Runx2 is an important transcription factor for differentiation of mesenchymal precursor cells into osteoblasts [39–41]. Therefore, we measured the effect of Dex on the Smad1/5/8 and Runx2 expressions during BMP-2 incubation in this study. The results indicated that in the cells incubated with (BMP-2 +Dex) or BMP-2, the protein levels of Id-1 and Smad1/5/8 had no significant difference, which means that the BMP-Smad signal channel may not be the reason for the high expression of ALP activity induced by the synergistic effect of Dex and BMP-2. But the level of Runx2 protein and mRNA expression of (BMP-2+Dex)-treatment were much higher than the cells induced by Dex or BMP-2 alone. Moreover, the *in vivo* Runx2 protein level of (BMP-2+Dex)-treatment was also higher than that only induced by BMP-2. The results imply that Runx2 may play a key role in the osteogenic differentiation of C2C12 cells induced by (Dex+BMP-2).

This noticeable difference with regard to Runx2 mRNA/protein levels between BMP-2 alone and BMP-2 + Dex-treatment arouse our attention. Treatment with BMP-2 not only inhibited the expression of C2C12 cells’ master genes (including MyoD) and blocked their myogenic differentiation, but also enhanced osteogenic differentiation by triggering the expression of osteogenic-related genes [42]. It was also reported that in C2C12 cells, the regulation of Runx2 played a key role in blocking the myogenic differentiation of C2C12 cells and the process of osteogenic differentiation induced by BMP-2 [43]. Taking into account these considerations, two explanations for the above stimulatory effect of Dex on the osteogenic differentiation induced by BMP-2 are possible.

The first possibility, we believe, is related to the suppression of the master-forming genes expression and thus the blockage of the formation of myoblast lineage. As a kind of skeletal muscle myogenic progenitor cell, C2C12 is thought to differentiate into muscle cells. But BMP-2 can reverse this process by up-regulating the expression of Id-1 and Runx2 and reducing the activity of MyoD protein. Overexpression of Runx2 can inhibit the activity of MyoD protein and prevent the myogenic differentiation of C2C12. Moreover, it has been documented that the inhibition of Runx2 gene in mice can lead to complete cessation of osteogenic differentiation [27]. Therefore, it can be hypothesized that up-regulation Runx2 triggered by Dex might potentiate the suppressing of the MyoD family and the inducing of Smad-Runx2 signaling pathway. As a result, the ALP activity, OC and Col I mRNA were all increased.

The second cause is associated with the role of BMP-activated Smads and Runx2 signal pathway. Although Runx2 plays a very important role in the process of osteogenic differentiation, there are many other factors in this process. For example, all phosphorylated Smad proteins (Smad 1/3/5/8) and Runx1, Runx2 and Runx3 all show interactions *in vitro*. It has been reported that Runx3 and Smad3 can regulate the TβRE gene together [44]. Lee *et al*. [43] also found that in C2C12 cells, the synergistic effect of BMP-activated Smads and Runx2 could induce the overexpression of its osteogenic-specific genes. Our experimental results also supported this hypothesis. The expression of Runx2 induced alone by Dex was not enough to induce the osteoblast differentiation. Only under the condition of BMP-2 co-cultivation, Dex can enhance the osteoblast differentiation. Therefore, we conclude, the combination of the up-regulation Runx2 and BMP-activated Smads stimulate the differentiation of C2C12.

**Figure 6.**
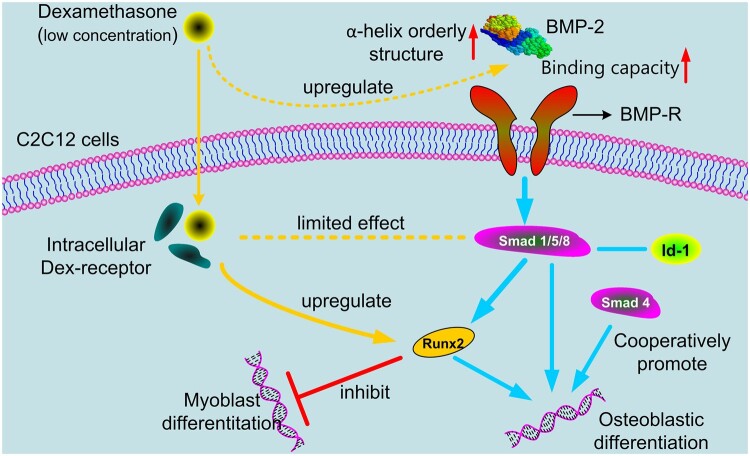
Cartoon model showing the regulation of Dex on the binding of BMP-2 to its receptor and BMP-2-induced osteoblastic differentiation via Runx2 activation. Dex at low concentration is beneficial to the ability of BMP-2 to bind to cell membrane surface receptors and thus up-regulated the downstream signaling pathway. Dex has no obvious effect on phospho-Smad1/5/8 protein and id-1, but it can up-regulate Runx2 expression and then promote osteoblastic differentiation and inhibit myoblast differentiation in C2C12 cell model

Taken together, the above results suggested that in C2C12 cells, 5–500 nM Dex could effectively enhance the binding of BMP-2 and BMP-R, and also enhance BMP-2-induced ALP expression, and (Dex+BMP-2)-treatment could significantly promote several osteoblast markers and osteogenic transcription factors mRNA expression. We also confirmed that Runx2 was involved in the synergistic osteogenic effects of Dex and BMP-2 both in C2C12 cells and *in vivo*. Previous studies have reported that the receptor site of Dex is in cytoplasm and the receptor of BMP-2 is on the surface of cell membrane. Therefore, based on these results obtained here and the previous reports, the mechanism model of Dex and BMP-2 synergistically inducing bone formation in the C2C12 cell model was proposed (Fig. 6). Firstly, low concentration of Dex facilitates recognition of BMP-2 with its receptors, and then the BMP-2 receptors form a heterodimer and initiate the signal transduction cascade by phosphorylating downstream cytosolic factors, like Smad 1/5/8. Secondly, the complex formed by Dex binding to its receptor, together with the Smad molecules, regulates the expression of downstream Runx2, which subsequently recognizes and binds to specific DNA sequence binding site, and then inhibits the myogenic differentiation and promotes the osteoblast differentiation. The C2C12 model and thigh muscle pouch implant model have demonstrated that Dex potentiates osteoblast differentiation induced by BMP-2 both *in vitro* and *in vivo*. With this respect, this study will lead us to find an optimal concentration ratio of Dex and BMP-2 for better osteogenic differentiation and will hasten the therapeutic application of a combined therapy of Dex and BMP-2.

## Conclusions

In this study, we identified that Dex with low concentration could significantly potentiate osteoblast differentiation induced by BMP-2 in C2C12 cells *in vitro* and the ectopic osteogenesis *in vivo*, as indicated by higher ALP levels, enhanced mineralization and better binding capacity to its receptors. It also appeared that compared with other experimental groups, the mRNA and protein expression of Runx2 increased significantly, which was consistent with the gene expression level of ALP. With these findings, it can be inferred that Dex at low concentrations may enhance the BMP-2 induced bone reconstruction, which may provide a new strategy to stimulate bone regeneration through the combined use of Dex and BMP-2. The results obtained here may be valuable for future research on the special signal transduction pathway of BMP-2 and the design of biomaterials for bone repair.

## Supplementary data

Supplementary data are available at *REGBIO* online.

## Funding

This work was supported by the Natural Science Foundation of China for Innovative Research Groups (No. 51621002), the National Natural Science Foundation of China (No. 32071337), Shanghai Pujiang Program (20PJ1402600) and the 111 Project (B14018). This study was also supported by ‘the Fundamental Research Funds for the Central Universities’.

*Conflict of interest statement*. None declared.

## Supplementary Material

rbac008_Supplementary_Data

## References

[1] Bolland B , Tilley S, New AMR, Dunlop DG, Oreffo ROC. Adult mesenchymal stem cells and impaction grafting: a new clinical paradigm shift. Expert Rev Med Devices 2007;4:393–404. 17488232 10.1586/17434440.4.3.393

[2] Freeman FE , Pitacco P, van Dommelen LHA, Nulty J, Browe DC, Shin J-Y, Alsberg E, Kelly DJ. 3D bioprinting spatiotemporally defined patterns of growth factors to tightly control tissue regeneration. Sci Adv 2020;6:5093. 10.1126/sciadv.abb5093 PMC7428335 32851179

[3] Herberg S , McDermott AM, Dang PN, Alt DS, Tang R, Dawahare JH, Varghai D, Shin JY, McMillan A, Dikina AD, He F, Lee YB, Cheng Y, Umemori K, Wong PC, Park H, Boerckel JD, Alsberg E. Combinatorial morphogenetic and mechanical cues to mimic bone development for defect repair. Sci Adv 2019;5:2476. 10.1126/sciadv.aax2476 PMC6713501 31489377

[4] McDermott AM , Herberg S, Mason DE, Collins JM, Pearson HB, Dawahare JH, Tang R, Patwa AN, Grinstaff MW, Kelly DJ, Alsberg E, Boerckel JD. Recapitulating bone development through engineered mesenchymal condensations and mechanical cues for tissue regeneration. Sci Transl Med 2019;11:7756. 10.1126/scitranslmed.aav7756 PMC6959418 31167930

[5] Chen D , Zhao M, Mundy GR. Bone morphogenetic proteins. Growth Factors 2004;22:233–41. 15621726 10.1080/08977190412331279890

[6] Lian H , Wang H, Han Q, Wang C. Quantification of rhBMP2 in bioactive bone materials. Regen Biomater 2020;7:71–6. 32153993 10.1093/rb/rbz038 PMC7053258

[7] Xu T , Sheng L, He L, Weng J, Duan K. Enhanced osteogenesis of hydroxyapatite scaffolds by coating with BMP-2-loaded short polylactide nanofiber: a new drug loading method for porous scaffolds. Regen Biomater 2020;7:91–8. 32440360 10.1093/rb/rbz040 PMC7233607

[8] Attisano L , Wrana JL. Signal transduction by the TGF-beta superfamily. Science 2002;296:1646–7. 12040180 10.1126/science.1071809

[9] Gan Q , Zhu JY, Yuan Y, Liu HL, Zhu YH, Liu CS. A proton-responsive ensemble using mesocellular foam supports capped with N, O-carboxymethyl chitosan for controlled release of bioactive proteins. J Mater Chem B 2015;3:2281–5. 32262057 10.1039/c5tb00219b

[10] Hettiaratchi MH , Krishnan L, Rouse T, Chou C, McDevitt TC, Guldberg RE. Heparin-mediated delivery of bone morphogenetic protein-2 improves spatial localization of bone regeneration. Sci Adv 2020;6:1240. 10.1126/sciadv.aay1240 PMC6941907 31922007

[11] Raina DB , Matuszewski L-M, Vater C, Bolte J, Isaksson H, Lidgren L, Tagil M, Zwingenberger S. A facile one-stage treatment of critical bone defects using a calcium sulfate/hydroxyapatite biomaterial providing spatiotemporal delivery of bone morphogenic protein-2 and zoledronic acid. Sci Adv 2020;6:1779. 10.1126/sciadv.abc1779 PMC7695465 33246951

[12] Zhang B , Skelly JD, Maalouf JR, Ayers DC, Song J. Multifunctional scaffolds for facile implantation, spontaneous fixation, and accelerated long bone regeneration in rodents. Sci Transl Med 2019;11:7411. 10.1126/scitranslmed.aau7411 31341064

[13] Chen X , Tan B, Bao Z, Wang S, Tang R, Wang Z, Chen G, Chen S, Lu WW, Yang D, Peng S. Enhanced bone regeneration via spatiotemporal and controlled delivery of a genetically engineered BMP-2 in a composite Hydrogel. Biomaterials 2021;277:121117. 34517277 10.1016/j.biomaterials.2021.121117

[14] Sanchez-Casanova S , Martin-Saavedra FM, Escudero-Duch C, Uceda MIF, Prieto M, Arruebo M, Acebo P, Fabiilli ML, Franceschi RT, Vilaboa N. Local delivery of bone morphogenetic protein-2 from near infrared-responsive hydrogels for bone tissue regeneration. Biomaterials 2020;241:119909. 32135355 10.1016/j.biomaterials.2020.119909 PMC7263445

[15] Lin D , Zhang J, Bai F, Cao XH, Fan CY, Yuan Y, Wang JW, Zhang J, Liu CS. Fabrication and clinical application of easy-to-operate pre-cured CPC/rhBMP-2 micro-scaffolds for bone regeneration. Am J Transl Res 2016;8:1379–96. 27186266 PMC4859626

[16] Kim HJ , Hong SJ, Lee S, Park JM, Park J-I, Park JS, Shim SH, Park K-H. Induction of bone formation by 3D biologically active scaffolds containing RGD-NPs, BMP2, and NtMPCs. Adv Therap 2021;4:2000245.

[17] Liu K , Meng C-X, Lv Z-Y, Zhang Y-J, Li J, Li K-Y, Liu F-Z, Zhang B, Cui F-Z. Enhancement of BMP-2 and VEGF carried by mineralized collagen for mandibular bone regeneration. Regen Biomater 2020;7:435–40. 32793388 10.1093/rb/rbaa022 PMC7414995

[18] Briquez PS , Tsai H-M, Watkins EA, Hubbell JA. Engineered bridge protein with dual affinity for bone morphogenetic protein-2 and collagen enhances bone regeneration for spinal fusion. Sci Adv 2021;7:4302. 10.1126/sciadv.abh4302 PMC8195475 34117071

[19] Johnson CT , Sok MCP, Martin KE, Kalelkar PP, Caplin JD, Botchwey EA, Garcia AJ. Lysostaphin and BMP-2 co-delivery reduces *S. aureus* infection and regenerates critical-sized segmental bone defects. Sci Adv 2019;5:1228. 10.1126/sciadv.aaw1228 PMC6524983 31114804

[20] Seeherman HJ , Berasi SP, Brown CT, Martinez RX, Juo ZS, Jelinsky S, Cain MJ, Grode J, Tumelty KE, Bohner M, Grinberg O, Orr N, Shoseyov O, Eyckmans J, Chen C, Morales PR, Wilson CG, Vanderploeg EJ, Wozney JM. A BMP/activin A chimera is superior to native BMPs and induces bone repair in nonhuman primates when delivered in a composite matrix. Sci Transl Med 2019;11:4953. 10.1126/scitranslmed.aar4953 31019025

[21] Liu H , Zhang H, Yin N, Zhang Y, Gou J, Yin T, He H, Ding H, Zhang Y, Tang X. Sialic acid-modified dexamethasone lipid calcium phosphate gel core nanoparticles for target treatment of kidney injury. Biomater Sci 2020;8:3871–84. 32519704 10.1039/d0bm00581a

[22] Mikami Y , Asano M, Honda MJ, Takagi M. Bone morphogenetic protein 2 and dexamethasone synergistically increase alkaline phosphatase levels through JAK/STAT signaling in C3H10T1/2 cells. J Cell Physiol 2010;223:123–33. 20039267 10.1002/jcp.22017

[23] Jager M , Fischer J, Dohrn W, Li XN, Ayers DC, Czibere A, Prall WC, Lensing-Hohn S, Krauspe R. Dexamethasone modulates BMP-2 effects on mesenchymal stem cells in vitro. J Orthop Res 2008;26:1440–8. 18404732 10.1002/jor.20565

[24] Holyoak DT , Wheeler TA, van der Meulen MCH, Singh A. Injectable mechanical pillows for attenuation of load-induced post-traumatic osteoarthritis. Regen Biomater 2019;6:211–9. 31402982 10.1093/rb/rbz013 PMC6683954

[25] Li L , Zhou GL, Wang Y, Yang G, Ding S, Zhou SB. Controlled dual delivery of BMP-2 and dexamethasone by nanoparticle-embedded electrospun nanofibers for the efficient repair of critical-sized rat calvarial defect. Biomaterials 2015;37:218–29. 25453952 10.1016/j.biomaterials.2014.10.015

[26] Zhou HJ , Qian JC, Wang J, Yao WT, Liu CS, Chen JG, Cao XH. Enhanced bioactivity of bone morphogenetic protein-2 with low dose of 2-N, 6-O-sulfated chitosan in vitro and in vivo. Biomaterials 2009;30:1715–24. 19131102 10.1016/j.biomaterials.2008.12.016

[27] Ruckh TT , Kumar K, Kipper MJ, Popat KC. Osteogenic differentiation of bone marrow stromal cells on poly(epsilon-caprolactone) nanofiber scaffolds. Acta Biomater 2010;6:2949–59. 20144747 10.1016/j.actbio.2010.02.006

[28] Jorgensen NR , Henriksen Z, Sorensen OH, Civitelli R. Dexamethasone, BMP-2, and 1,25-dihydroxyvitamin D enhance a more differentiated osteoblast phenotype: validation of an in vitro model for human bone marrow-derived primary osteoblasts. Steroids 2004;69:219–26. 15183687 10.1016/j.steroids.2003.12.005

[29] Mikami Y , Omoteyama K, Kato S, Takagi M. Inductive effects of dexamethasone on the mineralization and the osteoblastic gene expressions in mature osteoblast-like ROS17/2.8 cells. Biochem Biophys Res Commun 2007;362:368–73. 17707772 10.1016/j.bbrc.2007.07.192

[30] Martins A , Duarte ARC, Faria S, Marques AP, Reis RL, Neves NM. Osteogenic induction of hBMSCs by electrospun scaffolds with dexamethasone release functionality. Biomaterials 2010;31:5875–85. 20452016 10.1016/j.biomaterials.2010.04.010

[31] Walsh S , Jordan GR, Jefferiss C, Stewart K, Beresford JN. High concentrations of dexamethasone suppress the proliferation but not the differentiation or further maturation of human osteoblast precursors in vitro: relevance to glucocorticoid-induced osteoporosis. Rheumatology (Oxford) 2001;40:74–83. 11157145 10.1093/rheumatology/40.1.74

[32] Carpenter RS , Goodrich LR, Frisbie DD, Kisiday JD, Carbone B, McIlwraith CW, Centeno CJ, Hidaka C. Osteoblastic differentiation of human and equine adult bone marrow-derived mesenchymal stem cells when BMP-2 or BMP-7 homodimer genetic modification is compared to BMP-2/7 heterodimer genetic modification in the presence and absence of dexamethasone. J Orthop Res 2010;28:1330–7. 20309952 10.1002/jor.21126 PMC3200399

[33] Matsumoto Y , Otsuka F, Takano M, Mukai T, Yamanaka R, Takeda M, Miyoshi T, Inagaki K, Sada K, Makino H. Estrogen and glucocorticoid regulate osteoblast differentiation through the interaction of bone morphogenetic protein-2 and tumor necrosis factor-alpha in C2C12 cells. Mol Cell Endocrinol 2010;325:118–27. 20638987 10.1016/j.mce.2010.05.004

[34] Itoh S , Udagawa N, Takahashi N, Yoshitake F, Narita H, Ebisu S, Ishihara K. A critical role for interleukin-6 family-mediated Stat3 activation in osteoblast differentiation and bone formation. Bone 2006;39:505–12. 16679075 10.1016/j.bone.2006.02.074

[35] Hirano T , Ishihara K, Hibi M. Roles of STAT3 in mediating the cell growth, differentiation and survival signals relayed through the IL-6 family of cytokine receptors. Oncogene 2000;19:2548–56. 10851053 10.1038/sj.onc.1203551

[36] Ishihara K , Hirano T. Molecular basis of the cell specificity of cytokine action. BBA Mol Cell Res 2002;1592:281–96. 10.1016/s0167-4889(02)00321-x 12421672

[37] Jun JH , Yoon WJ, Seo SB, Woo KM, Kim GS, Ryoo HM, Baek JH. BMP2-activated Erk/MAP kinase stabilizes Runx2 by increasing p300 levels and histone acetyltransferase activity. J Biol Chem 2010;285:36410–9. 20851880 10.1074/jbc.M110.142307 PMC2978570

[38] Wang Q , Wei XC, Zhu TH, Zhang M, Shen R, Xing LP, O'Keefe RJ, Chen D. Bone morphogenetic protein 2 activates Smad6 gene transcription through bone-specific transcription factor Runx2. J Biol Chem 2007;282:10742–8. 17215250 10.1074/jbc.M610997200 PMC2636961

[39] Jeon EJ , Lee KY, Choi NS, Lee MH, Kim HN, Jin YH, Ryoo HM, Choi JY, Yoshida M, Nishino N, Oh BC, Lee KS, Lee YH, Bae SC. Bone morphogenetic protein-2 stimulates Runx2 acetylation. J Biol Chem 2006;281:16502–11. 16613856 10.1074/jbc.M512494200

[40] Phillips JE , Gersbach CA, Wojtowicz AM, Garcia AJ. Glucocorticoid-induced osteogenesis is negatively regulated by Runx2/Cbfa1 serine phosphorylation. J Cell Sci 2006;119:581–91. 16443755 10.1242/jcs.02758

[41] Zhang YY , Li X, Qian SW, Guo L, Huang HY, He Q, Liu Y, Ma CG, Tang QQ. Down-regulation of type I Runx2 mediated by dexamethasone is required for 3T3-L1 adipogenesis. Mol Endocrinol 2012;26:798–808. 22422618 10.1210/me.2011-1287 PMC5417096

[42] Kawaski T , Niki Y, Miyamoto T, Horiuchi K, Matsumoto M, Aizawa M, Toyama Y. The effect of timing in the administration of hepatocyte growth factor to modulate BMP-2-induced osteoblast differentiation. Biomaterials 2010;31:1191–8. 19913294 10.1016/j.biomaterials.2009.10.048

[43] Lee KS , Kim HJ, Li QL, Chi XZ, Ueta C, Komori T, Wozney JM, Kim EG, Choi JY, Ryoo HM, Bae SC. Runx2 is a common target of transforming growth factor beta 1 and bone morphogenetic protein 2, and cooperation between Runx2 and Smad5 induces osteoblast-specific gene expression in the pluripotent mesenchymal precursor cell line C2C12. Mol Cell Biol 2000;20:8783–92. 11073979 10.1128/mcb.20.23.8783-8792.2000 PMC86511

[44] Hanai J , Chen LF, Kanno T, Ohtani-Fujita N, Kim WY, Guo WH, Imamura T, Ishidou Y, Fukuchi M, Shi MJ, Stavnezer J, Kawabata M, Miyazono K, Ito Y. Interaction and functional cooperation of PEBP2/CBF with Smads - Synergistic induction of the immunoglobulin germline C alpha promoter. J Biol Chem 1999;274:31577–82. 10531362 10.1074/jbc.274.44.31577

