# Supplementary material for: Fabrication and evaluation of a BMP-2/dexamethasone co-loaded gelatin sponge scaffold for rapid bone regeneration
Source: Regen Biomater. 2022 Feb 16;9:rbac008. doi: 10.1093/rb/rbac008 (PMC9113239; doi:10.1093/rb/rbac008)
Supplement: rbac008_Supplementary_Data [file rbac008_supplementary_data.docx]

**Supporting Information**

Fabrication and evaluation of a BMP-2/dexamethasone co-loaded gelatin sponge scaffold for rapid bone regeneration

Qi Gan, ^a, b^, Hao Pan, ^c^ Wenjing Zhang, ^c^ Yuan Yuan, *^, a^ Jiangchao Qian, ^b^ Changsheng Liu **^, a, c, d^

^a^ Key Laboratory for Ultrafine Materials of Ministry of Education, East China University of Science and Technology, Shanghai, 200237, PR China

^b^ The State Key Laboratory of Bioreactor Engineering, East China University of Science and Technology, Shanghai, 200237, PR China

^c^ Engineering Research Center for Biomedical Materials of the Ministry of Education, East China University of Science and Technology, Shanghai, 200237, PR China

^d^ Frontiers Science Center for Materiobiology and Dynamic Chemistry, East China University of Science and Technology, Shanghai, 200237, PR China

**
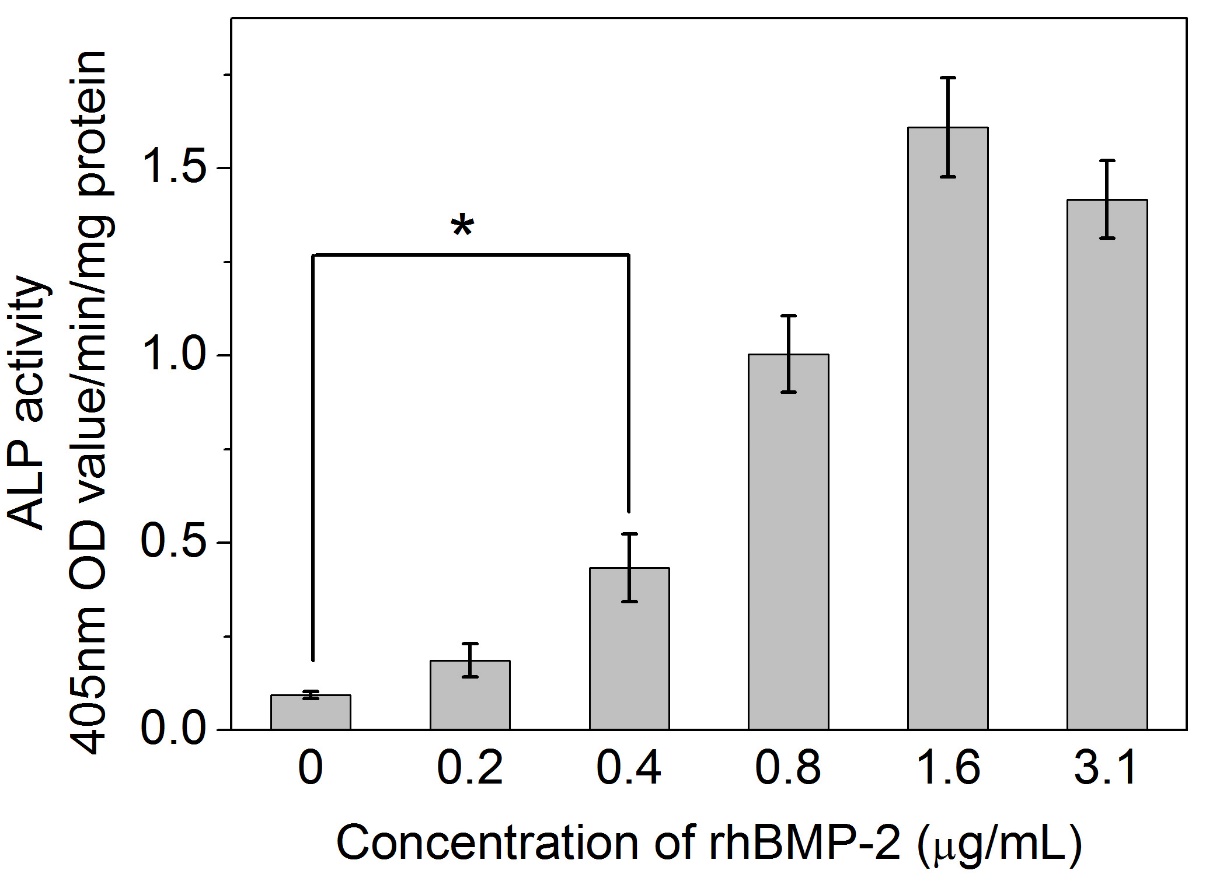
**

**Figure S1.** Effects of BMP-2-treatment alone on ALP activity in C2C12 cells. Cells were cultured with graded concentrations of BMP-2 for 3 days. ALP was measured at day 3 using soluble substrate p-nitrophenylphosphate. The values represent the mean ± standard deviation (n = 4). *p < 0.05.

**
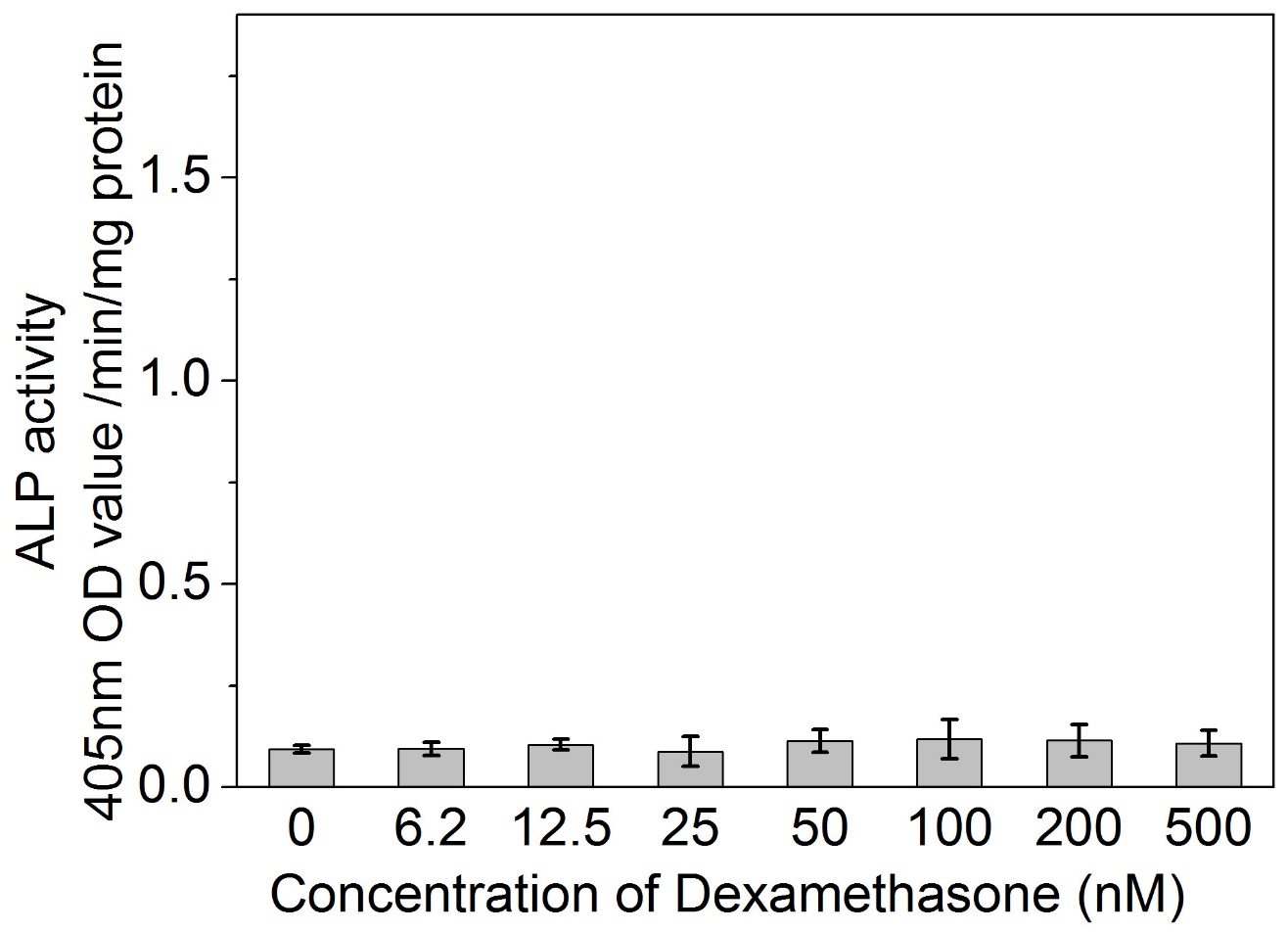
**

**Figure S2.** Effects of dexamethasone-treatment alone on ALP activity in C2C12 cells. Cells were cultured with graded concentrations of dexamethasone for 3 days. ALP was measured at day 3 using soluble substrate p-nitrophenylphosphate. The values represent the mean ± standard deviation (n = 4). *p < 0.05.
